# Enhancement by *Burkholderia contaminans* ZCC Combined with Biochar on the Remediation of Arsenic-Contaminated Soil by *Pteris vittata*

**DOI:** 10.3390/plants14203169

**Published:** 2025-10-15

**Authors:** Xiaojun Yang, Yuanping Li, Dan Zhou, Hend Alwathnani, Christopher Rensing

**Affiliations:** 1 Fujian Provincial Key Laboratory of Eco-Industrial Green Technology, College of Ecology and Resources Engineering, Wuyi University, Wuyishan 354300, China; yangxiaojun0828@163.com; 2 College of Tea and Food, Wuyi University, Wuyishan 354300, China; li343000@126.com; 3 Institute of Environmental Microbiology, Fujian Agriculture and Forestry University, Fuzhou 350002, China; a33161500@126.com; 4 Department of Botany and Microbiology, King Saud University, Riyadh 11451, Saudi Arabia; wathnani@ksu.edu.sa

**Keywords:** *Burkholderia contaminans*, biochar, phytoremediation, *Pteris vittata*, soil remediation

## Abstract

Arsenic pollution is a global environmental challenge, necessitating efficient and sustainable remediation technologies. This study investigates the synergistic effect of the arsenic-resistant bacterium *Burkholderia contaminans* ZCC (ZCC) and corn stalk biochar (BC) on arsenic-contaminated soil, with *Pteris vittata* as the remediation plant. Through pot experiments, we evaluated the effects of various BC addition rates (0%, 1%, 5%) and ZCC inoculation on soil pH, plant growth, physiological responses, and arsenic accumulation. Biochar alone significantly increased soil pH (reaching 7.56 in the 5% BC treatment), while *B. contaminans* ZCC alone had a weaker effect. In combined treatments, pH changes were primarily driven by biochar. The combination of *B. contaminans* ZCC and BC enhanced *P. vittata* growth, with the 5% BC + ZCC treatment showing the greatest increase in total plant biomass (2.56 times that of the control) and total chlorophyll content (43.32% higher). This treatment also activated antioxidant systems (increased SOD, POD, and CAT activities), reduced oxidative damage (lower MDA content), and improved osmotic regulation (higher proline content). Notably, *B. contaminans* ZCC and BC synergistically enhanced arsenic accumulation in the *P. vittata* plant, with the arsenic content under the 5% BC + ZCC treatment being 2.81 times that of the control. This study demonstrates that the combination of *B. contaminans* ZCC and BC enhances arsenic remediation through soil improvement and plant growth promotion.

## 1. Introduction

Heavy metal(loid) pollution in soil has become a global environmental issue, with arsenic contamination receiving particular attention due to its strong toxicity and high mobility. Biochar, recognized for its excellent specific surface area, strong adsorption capacity, and significant cation exchange potential, is considered a highly promising material for remediating heavy metal-contaminated soils [1]. Additionally, biochar is primarily derived from agricultural and forestry waste, a characteristic that enables the utilization of waste while simultaneously repairing the soil, garnering widespread attention in the academic community [2]. Phytoremediation technology, as a low-cost, low-interference, and secondary pollution-free soil remediation strategy, has become one of the key approaches for addressing heavy metal pollution issues. Among numerous hyperaccumulators, *Pteris vittata* has emerged as an ideal candidate for the remediation of arsenic-contaminated soils due to its exceptional ecological adaptability, stress resistance, rapid growth rate, and efficient arsenic hyperaccumulation capacity [3,4].

Although both biochar and *P. vittata* have demonstrated significant potential in arsenic contamination remediation, the synergistic effects and underlying mechanisms of their combined application require further in-depth investigation [5]. The application of biochar has been shown to influence the absorption and translocation of arsenic by *P. vittata* by altering soil physicochemical properties (such as pH and redox potential) as well as the speciation and bioavailability of soil arsenic [5,6].

Furthermore, rhizosphere microorganisms play a pivotal role in promoting plant growth and development, enhancing plant stress resistance, and improving the efficiency of heavy metal remediation. *Burkholderia* is a common genus among rhizosphere microorganisms, exhibiting significant growth-promoting effects and serving as an important microbial resource for the biological control of plant diseases [7].

This study aimed to investigate the combined effects of biochar and phytoremediation, as well as microbial-assisted remediation, on arsenic-contaminated soil. Specifically, artificially prepared arsenite-contaminated soil was used as the test subject, with the arsenic hyperaccumulator *P. vittata* selected as the remediation plant, combined with biochar as an amendment. On this basis, *Burkholderia contaminans* ZCC, previously isolated from the soil of Zijinshan gold-copper mine by our research group, was introduced. This strain exhibits heavy metal(loid) resistance (with a minimum inhibitory concentration of 6 mM for arsenite) and plant growth-promoting traits (capable of producing indole acetic acid (IAA), siderophores, and 1-aminocyclopropane-1-carboxylate (ACC) deaminase) [8,9]. Through plant pot experiments, this study delved into the effects of corn stalk biochar (BC) addition on the remediation of arsenic-contaminated soil by *P. vittata* from the soil-plant system level, as well as the influence of *B. contaminans* ZCC on arsenic uptake by *P. vittata* and the phytoremediation efficacy. The research findings will provide a reference basis for the theoretical research and practical application of biochar, *P. vittata*, and *B*. *contaminans* ZCC in the combined remediation of arsenic-contaminated soil, and lay the foundation for subsequent field remediation trials.

## 2. Results

### 2.1. Effects of B. contaminans ZCC and Biochar Co-Application on Soil pH

Soil pH is a key factor regulating the transformation of heavy metals, and an increase in soil pH has been shown to promote the formation of hydroxide precipitates or iron and manganese oxide-bound states of metal(loid)s such as arsenic, thereby reducing their bioavailability [10]. As shown in Table 1, compared to the control group (CK), the application of the arsenic-resistant *B*. *contaminans* ZCC alone significantly increased soil pH (*p* < 0.05). The 1% and 5% BC treatments raised the pH by 5.2% and 12.31%, respectively (*p* < 0.05). While the combined application of *B*. *contaminans* ZCC and biochar also significantly increased pH compared to CK, there was no significant difference between the combined treatments and the respective biochar treatments alone (*p* > 0.05).

### 2.2. Effects of B. contaminans ZCC and Biochar Co-Application on the Growth of P. vittata

As shown in Table 2, the application of strain ZCC or biochar alone, as well as the combined application of *B. contaminans* ZCC and biochar, all resulted in increases in the total plant fresh weight, total plant dry weight, and height of *P. vittata* to varying degrees. The total plant biomass (fresh weight and dry weight) and height of *P. vittata* treated with only strain ZCC were increased by 36.48%, 24.74%, and 15.53%, respectively, compared to CK. When *B. contaminans* ZCC was combined with 5% biochar, the biomass of *P. vittata* reached its maximum, with total plant fresh weight, total plant dry weight, and height being 2.56, 2.31, and 1.48 times that of CK, respectively.

### 2.3. Effects of B. contaminans ZCC and Biochar Co-Application on Chlorophyll Content of P. vittata

**Figure 1:**
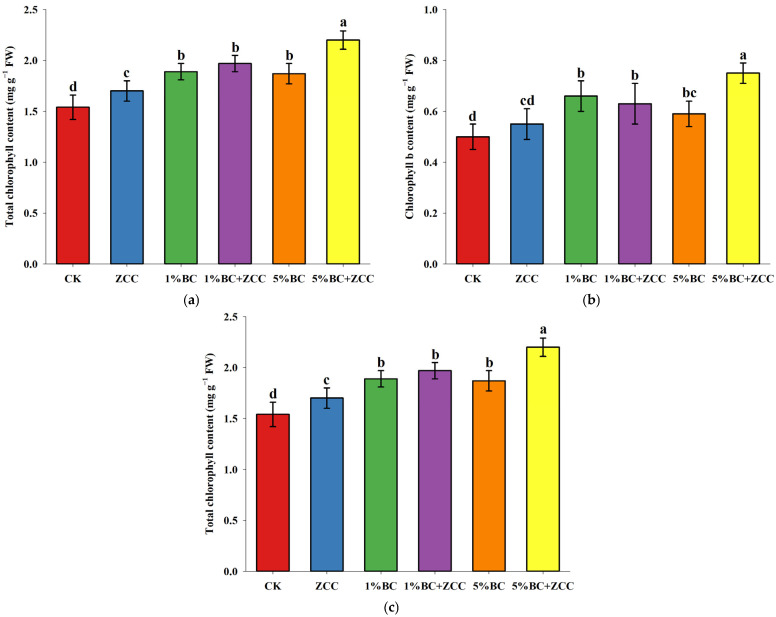
Changes in chlorophyll content of *P. vittata*. (**a**) chlorophyll a content; (**b**) chlorophyll b content; (**c**) total chlorophyll content. The data displays means (*n* = 6) and different letters with means show significance with ± SD at *p <* 0.05 (Duncan’s new multiple range test).

As shown in Figure 1, different treatments had a significant impact on the chlorophyll content of *P. vittata*. The results indicated that the application of different ratios of biochar alone, as well as the combined application of biochar and strain ZCC, significantly increased the total chlorophyll, chlorophyll a, and chlorophyll b contents in the leaves of *P. vittata* exposed to arsenic stress. Among these treatments, the 5% BC + ZCC treatment displayed the most significant effect, with total chlorophyll, chlorophyll a, and chlorophyll b contents increasing by 43.32%, 46.28%, and 48.50%, respectively.

### 2.4. Effects of B. contaminans ZCC and Biochar Co-Application on Antioxidant Enzyme Activity in P. vittata Roots

**Figure 2:**
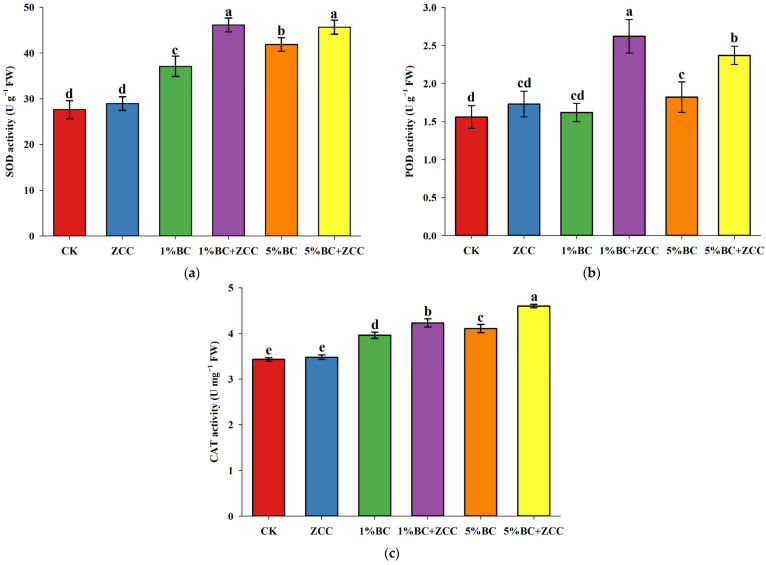
Changes in antioxidant enzyme activity in *P. vittata* roots. (**a**) SOD; (**b**) POD; (**c**) CAT. The data displays means (*n* = 6) and different letters with means show significance with ± SD at *p* < 0.05 (Duncan’s new multiple range test).

As shown in Figure 2a, increasing biochar application significantly enhanced the superoxide dismutase (SOD) activity in *P. vittata* roots. The strain ZCC-only treatment showed no significant effect on SOD activity compared to CK. However, the combined treatments with biochar (1% BC + ZCC, 5% BC + ZCC) significantly increased SOD activity, with increases of 59.35% and 57.62%, respectively, compared to the ZCC-only treatment. These results suggest that biochar effectively enhances SOD activity in *P. vittata* roots, helping to alleviate arsenic-induced stress. As shown in Figure 2b, peroxidase (POD) activity in *P. vittata* roots increased with the application of biochar. While the application of strain ZCC alone or a low dose (1%) of biochar resulted in no significant difference in POD activity. However, the application of a higher dose (5%) of biochar alone and the combined application of biochar with strain ZCC significantly enhanced POD activity, with the 1% BC + ZCC treatment showing the highest increase, 68.17% higher than CK. These results suggest that the co-application of biochar and the arsenic-resistant bacterium *B*. *contaminans* ZCC significantly enhances the POD activity in *P. vittata* roots, improving the plant’s capacity to remediate arsenic-contaminated soils. As shown in Figure 2c, the application of strain ZCC alone had no significant effect on the catalase (CAT) activity in *P. vittata* roots. However, the BC + ZCC treatments significantly increased the CAT activity in *P. vittata* roots, particularly in the 5% BC + ZCC treatment, where CAT activity was the highest, increasing by 34.21% compared to CK.

### 2.5. Effects of B. contaminans ZCC and Biochar Co-Application on MDA and Proline Content in P. vittata Roots

**Figure 3:**
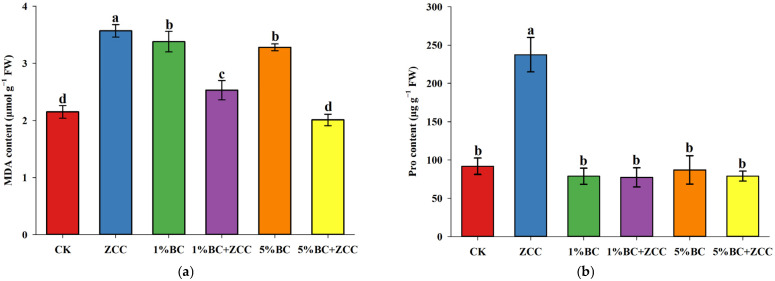
Changes in MDA content (**a**) and proline content (**b**) in *P. vittata* roots. The data is means (*n =* 6) and different letters with means show significance with ± SD at *p <* 0.05 (Duncan’s new multiple range test).

Compared to CK, the addition of the arsenic-resistant bacterium ZCC significantly increased the MDA content in *P. vittata* roots, indicating that the addition of strain ZCC had a certain impact on the cell membranes of *P. vittata*. Compared to the ZCC treatment, the application of different ratios of biochar alone, as well as the combined application with the growth-promoting bacterium *B*. *contaminans* ZCC, significantly reduced MDA content in the roots of *P. vittata*, with the 1% BC + ZCC and 5% BC + ZCC treatments showing reductions of 29.35% and 43.76%, respectively. However, the 5% BC + ZCC group showed no significant difference compared to CK (Figure 3a).

As shown in Figure 3b, the proline (Pro) content in *P. vittata* roots was highest under the ZCC treatment, showing a significant increase of 159.09% compared to CK, indicating that *B*. *contaminans* ZCC effectively induced proline biosynthesis of *P. vittata* to cope with arsenic stress.

### 2.6. Effects of B. contaminans ZCC and Biochar Co-Application on Arsenic Content in P. vittata Plant

As shown in Table 3, in the ZCC-only treatment group, the arsenic content in *P. vittata* was 22.42 mg kg^−1^, representing a 41.63% increase compared to CK. This indicates that strain ZCC can promote arsenic accumulation in *P. vittata*. Compared to CK, the arsenic content in *P. vittata* significantly increased after the application of 1% and 5% biochar (BC), with increases of 69.73% and 133.88%, respectively. This suggests that the addition of strain ZCC enhances the ability of biochar to promote arsenic accumulation in *P. vittata*. When strain ZCC was combined with 1% and 5% biochar, the arsenic content in *P. vittata* was significantly higher than with biochar applied alone, being 2.43 and 2.81 times that of CK, respectively.

## 3. Discussion

Biochar toxicity has been evaluated in recent work, supporting its safe use in remediation experiments [11]. Biochar accumulates alkaline substances, such as carbonates and metal oxides, during pyrolysis, thereby directly increasing soil alkalinity. The pyrolysis temperature and raw material type jointly determine the content of alkaline substances, and higher application rates (5% BC) resulted in a greater increase in pH [12]. *B*. *contaminans* ZCC may neutralize H^+^ by secreting alkaline metabolic products (such as ammonia and organic acid anions) and may activate microbial-mediated mineral weathering to release basic ions [13]. However, its effect on pH is weaker than that of biochar, indicating that the changes in pH in the combined treatment are primarily caused by biochar.

*B*. *contaminans* ZCC may promote root growth by secreting IAA and siderophores, thereby alleviating arsenic toxicity stress and increasing the biomass of *P. vittata* [14]. At the same time, biochar can retain and slowly release nutrients such as N and P, which can improve soil physicochemical properties and fertility, providing the necessary nutrients for *P. vittata* growth [15,16]. Research data indicate that the addition of the arsenic-resistant *B*. *contaminans* ZCC effectively reduced the toxicity of arsenic pollution in the soil, thus stimulating the growth of *P. vittata* in arsenic-contaminated soils. Further studies have shown that the combined application of the growth-promoting bacterium *B*. *contaminans* ZCC and biochar strengthened the growth-promoting effect of ZCC, significantly enhancing the tolerance and growth performance of *P. vittata*. Biochar, as a carrier, may extend the survival time of growth-promoting bacteria in the soil, enhance their resistance under heavy metal stress, and may synergistically promote plant fresh weight and growth through the release of small amounts of nutrients and improvement of soil physicochemical properties [17].

Chlorophyll, as a key pigment in plant photosynthesis, is an important physiological indicator reflecting the intensity of photosynthesis. During the photosynthetic process, chlorophyll a is primarily responsible for light energy conversion, while chlorophyll b mainly absorbs light energy [18]. Numerous studies have shown that heavy metal stress significantly inhibits the activity of key enzymes in the chlorophyll biosynthesis pathway while inducing the expression of chlorophyll-degrading enzymes (such as chlorophyllase), leading to an imbalance in the chlorophyll a/b ratio and a decrease in total chlorophyll content, thereby inhibiting the photochemical efficiency of photosystem II (PSII). This photosynthetic damage is an important mechanism by which heavy metal pollution reduces plant biomass [19]. Research indicates that biochar is able to adsorb nutrient elements in soil solution and reduce nutrient loss by improving soil water retention capacity, thus acting as a slow-release carrier for nutrient elements in the soil, helping to regulate soil nutrient cycling and maintain soil fertility [20,21]. The synergistic effect of *B*. *contaminans* ZCC and biochar not only enhanced the nutrient levels in the soil but also improved the growth environment for plants, promoting chlorophyll synthesis and resulting in leaves that are vibrant and glossy [22].

Under stress conditions, plants accumulate excessive reactive oxygen species (ROS), leading to oxidative damage. To resist such damage, plants have evolved an antioxidant defense system composed of core enzymes such as SOD, POD, and CAT, which work together to eliminate ROS and maintain redox homeostasis. SOD catalyzes the dismutation of superoxide radicals (O_2_•^−^) into H_2_O_2_, while POD and CAT further decompose H_2_O_2_ into H_2_O and O_2_, forming a complete ROS elimination pathway [23]. In this study, we found that as the proportion of biochar increased, the activities of SOD, POD, and CAT increased with the increasing application of biochar. Compared to the application of ZCC alone, the combination of ZCC and biochar significantly enhanced the activities of SOD, POD, and CAT in *P. vittata*. This indicates that the combined use of the arsenic-resistant bacterium ZCC and biochar can effectively enhance the antioxidant enzyme activity of *P. vittata*, thereby alleviating the stress caused by arsenic. Biochar may enhance ROS elimination through two pathways: the first is by directly providing redox-active sites; the surface functional groups of biochar (such as quinones) participate in electron transfer, reducing ROS accumulation [24]; the second is by indirectly regulating the rhizosphere environment: increasing soil pH and organic carbon content, promoting the proliferation and metabolic activity of ZCC, thereby enhancing the plant’s antioxidant response [5]. Thus, the combined application of biochar and plant growth-promoting bacteria can enhance plant stress resistance by increasing antioxidant enzyme activity. These three antioxidant enzymes work synergistically to form an effective antioxidant defense system, capable of eliminating excess superoxide radicals in the plant body, protecting the plant from oxidative damage, and further promoting dry matter accumulation.

MDA, as a key end product of lipid peroxidation, can directly reflect the extent of oxidative damage to cell membranes and is an important indicator for evaluating plant responses to abiotic stress [25]. Compared to the arsenic stress control group, the addition of *B*. *contaminans* ZCC alone significantly increased the MDA content in the roots of *P. vittata* (*p <* 0.05), indicating that under conditions without the synergistic effect of biochar, ZCC may have exacerbated the oxidative stress response in *P. vittata*. This phenomenon suggests that the strain activated oxidative pathways within the plant through unclear mechanisms (such as metabolic load or the production of harmful metabolites), leading to increased damage to the membrane system. The application of biochar alone also failed to alleviate this and instead exacerbated oxidative stress, which may be due to the physicochemical properties of biochar itself. On one hand, the application of biochar may alter the soil pH and redox potential, thereby affecting the chemical forms and bioavailability of arsenic, and even increasing the concentration of soluble arsenic under certain conditions, exacerbating toxicity to plants [5]. On the other hand, some biochar surfaces contain persistent free radicals (PFRs), which have been shown to catalyze the generation of highly reactive ROS such as hydroxyl radicals (·OH) from molecular oxygen or peroxides in the soil under specific conditions, causing direct oxidative damage to plant roots [26,27]. When *B*. *contaminans* ZCC was applied in combination with biochar, the oxidative stress in the roots of *P. vittata* was significantly alleviated. In contrast to the “stress overlay” effect of ZCC applied alone, the MDA content in the 1% BC + ZCC and 5% BC + ZCC treatment groups decreased by 29.35% and 43.76%, respectively, compared to the ZCC group. Notably, the MDA content in the 5% BC + ZCC group returned to a level that was not significantly different from that of the CK control group, indicating a strong positive synergistic effect between biochar and *B*. *contaminans* ZCC. This might have been due to biochar having a rich porous structure and a large specific surface area, providing an ideal colonization “microhabitat” or “refuge” for *B*. *contaminans* ZCC [28]. This physical isolation not only protects ZCC from competition and predation by other microorganisms in the soil but also, more importantly, may regulate the growth microenvironment of ZCC (such as moisture and nutrient supply), keeping it in a more favorable physiological metabolic state for the plant, thereby reducing the aforementioned potential negative effects [29,30]. At the same time, the strong adsorption capacity of biochar has been shown to act as a “buffer,” adsorbing and fixing any harmful secondary metabolites produced by ZCC, preventing them from directly contacting the plant roots [31].

Proline, as a key multifunctional amino acid, plays multiple roles in the plant response to abiotic stress. It not only maintains cell turgor pressure by regulating osmotic potential and protects subcellular structures and membrane integrity, but also acts as an efficient ROS scavenger, inhibiting oxidative damage and cell apoptosis, thereby enhancing plant stress tolerance [32]. The treatment with ZCC significantly increased proline content, which is consistent with the mechanism by which growth-promoting bacteria (PGPR) enhance plant stress tolerance. *B*. *contaminans* ZCC may stimulate the accumulation of osmotic protective substances by promoting the expression of proline synthesis genes or improving nitrogen metabolism, thereby alleviating arsenic-induced oxidative stress [33]. The biochar group displayed no significant changes, possibly because biochar mainly affects the bioavailability of arsenic through physicochemical pathways (such as increasing soil pH and organic carbon) rather than directly regulating proline metabolism [5]. Additionally, the interaction between ZCC and biochar may lead to accelerated proline degradation or improved ROS scavenging efficiency, reducing the need for proline accumulation [34].

In an acidic arsenic-contaminated soil environment, the application of peanut straw biochar or rapeseed straw biochar has been shown to effectively reduce the proline levels in soybean plants and significantly enhance their absorption capacity of mineral elements (such as phosphorus, potassium, etc.) [35]. The combined application of rice straw biochar and silicon not only substantially decreased the proline content in rice but also strengthened the activity of its antioxidant enzyme system, thereby effectively alleviating the oxidative stress damage induced by arsenic contamination [36]. These results indicate that under arsenic stress conditions, the application of biochar is able to significantly reduce the accumulation of proline in certain plants, although this phenomenon has not yet been confirmed by related studies in *P*. *vittata.*

Under heavy metal(loid) stress, plants accelerate their physiological and biochemical activities to produce abundant metabolites that complex with heavy metal(loid)s for detoxification. However, this process can also lead to increased heavy metal uptake by the plant, resulting in harmful toxic effects. As a “model” species for arsenic hyperaccumulation, *P. vittata* has been shown to have a strong ability to absorb, transport, and accumulate arsenic [37]. In comparison to the 1% application rate, the 5% application rate of biochar exhibited a significantly greater effect on the accumulation of arsenic in *P. vittata*. This enhancement is attributed to the presence of nutrients such as nitrogen (N) and phosphorus (P) in biochar, which function as fertilizers, promoting plant growth and consequently increasing the plant’s capacity for arsenic absorption from the soil [15,16]. At the same time, PGPR can secrete IAA and siderophore utilized by plants to promote root growth, alleviating the arsenic stress on the plant roots and increasing the biomass of *P. vittata* [14,38].

## 4. Materials and Methods

### 4.1. Soil and Plant Preparation

The soil used in the experiment was collected from a paddy field in Fuzhou, Fujian Province. To prevent the influence of surface vegetation on the microbial community in the soil, the top 5 cm of soil was removed during collection. After the soil was collected, it was naturally air-dried, ground, sieved through a 2 mm mesh, and stored for later use. The basic physicochemical properties of the test soil, including pH, organic matter content, total nitrogen, available phosphorus, and available potassium, are summarized in Table 4. These values provide the baseline conditions for subsequent plant–soil interaction experiments. Three kilograms (3.0 kg) of reserved soil was weighed out into polyvinyl chloride (PV) pots, and arsenite (NaAsO_2_) solution was added to achieve final arsenic mass fractions of 250 mg kg^−1^ in the soil. The concentration of arsenic added was adjusted according to the levels employed by Li et al. [39]. Different doses of corn straw biochar (0%, 1%, and 5%, w:w) were respectively added. Base fertilizers were applied with NH_4_NO_3_, KH_2_PO_4_, and KCl at rates of 100, 100, and 150 mg kg^−1^ for N, P, and K, respectively. After thoroughly mixing the base fertilizers, biochar, and soil, the pots were placed in a dark place for one month of aging treatment, with six replicates per group. The gametophytes of *P. vittata* were cultivated to develop 3–5 fronds following the method described by Hua et al. [40].

### 4.2. Bacterial Inoculum Preparation

Using an inoculation loop, *B*. *contaminans* ZCC stored in a −80 °C freezer was transferred to LB agar medium, inverted, and incubated at 28 °C. When single colonies appeared, a single colony was picked and transferred to LB liquid medium. The cultured strain was then shaken and incubated at 28 °C, 180 rpm for approximately 16 h. After centrifuging the bacterial suspension at 6000 rpm, 4 °C for 10 min, the supernatant was discarded, and the bacterial pellet was resuspended in 10 mM MgCl_2_. The bacterial suspension was diluted with 10 mM MgCl_2_ to an OD_600_ of 1.0 for subsequent inoculation experiments.

### 4.3. Biochar Preparation

The biochar was obtained from corn straw. Briefly, three times-washed corn straw with deionized water was air-dried at room temperature. Air-dried corn straw was subjected to pyrolysis at 450 °C for approximately 2 h in a muffle furnace under an oxygen-limited atmosphere. The produced biochar was ground and sieved to <2 mm, rinsed thoroughly with deionized water, and subsequently oven-dried at 80 °C. The final biochar was preserved in a desiccated glass vessel until use.

### 4.4. Greenhouse Pot Experiment with P. vittata

*P. vittata* seedlings with similar growth were randomly selected and transplanted into PV pots, with three seedlings planted per pot and six replicates per group were maintained. They were cultivated in a greenhouse at a temperature of 25 °C at a relative humidity of 75%, and a light cycle of 12 h/12 h light-dark alternation, with a light intensity of 350 μmol m^−2^ s^−1^. The experimental setup included six groups: (1) CK (Control group, As-contaminated soil without strain ZCC and biochar added), (2) ZCC (As-contaminated soil supplied with strain ZCC), (3) 1% BC (As-contaminated soil supplied with 1% corn straw biochar), (4) 1% BC + ZCC (As-contaminated soil supplied with 1% corn straw biochar and strain ZCC), (5) 5% BC (As-contaminated soil supplied with 5% corn straw biochar), (6) 5% BC + ZCC (As-contaminated soil supplied with 5% corn straw biochar and strain ZCC). All treatments were conducted under arsenic-contaminated soil conditions (250 mg/kg As), and no arsenic-free control was included in this study. After the seedlings stabilized, 30 mL of the arsenic-resistant bacterial suspension *B*. *contaminans* ZCC was added via root irrigation, while the control group was supplemented with 30 mL of 10 mM MgCl_2_. During this period, deionized water was applied to the soil every two days to maintain a soil moisture content of 70%.

### 4.5. Soil pH Measurement

After 45 days of growth, the *P. vittata* plants and soil were carefully separated. The rhizosphere soil was collected using the root-shaking method and placed in a well-ventilated area to air-dry naturally. Once air-dried, the soil was sieved through a 1 mm mesh. The processed soil samples were then placed in sterile plastic sealable bags, ensuring the bags were tightly sealed to prevent contamination or influence from external factors. A ten-gram sample of the air-dried and sieved soil was weighed and placed in a 50 mL beaker, mixed with 25 mL of deionized water. After thorough stirring for 5 min, the mixture was allowed to stand for 30 min. The pH of the supernatant was then measured using a PHS-3C pH meter (INESA Scientific Instrument, Shanghai, China).

### 4.6. Plant Growth Analysis

After 45 days of cultivation, two *P. vittata* plants were randomly selected from each pot, carefully washed with ultrapure water, and surface moisture was absorbed using filter paper. The plant height of each *P. vittata* was measured separately, and the total plant fresh weight of each plant was determined using an analytical balance. The plants were then placed in an oven at 105 °C for 30 min for enzyme inactivation, followed by drying at 70 °C until a constant weight was achieved, and then the total plant dry weight was measured.

### 4.7. Chlorophyll Content Determination

Referring to the method of Huang et al. [41], 0.5 g of representative leaves were selected from each treatment, cut into pieces, and placed in centrifuge tubes containing 25 mL of 80% (*v*/*v*) acetone, then incubated in the dark for 24 h. The absorbance of the extracts at 645 nm and 663 nm was measured using a Lambda 350 UV-Vis spectrophotometer (PerkinElmer, Waltham, MA, USA), and the chlorophyll a content, chlorophyll b content, and total chlorophyll content of the leaves were calculated according to the formula.

### 4.8. Antioxidant Enzyme Activity, MDA and Proline Content Assays

The activity of SOD was determined using the nitroblue tetrazolium photoreduction method; the activity of POD was measured by the guaiacol method; and the activity of CAT was assessed via the ultraviolet absorption method [42]. The content of MDA was determined using the thiobarbituric acid method, and the content of proline was measured by the sulfosalicylic acid method [42].

### 4.9. Arsenic Quantification in P. vittata Plant

To determine arsenic content in *P. vittata*, 0.2 g of dry plant tissue was accurately weighed into a digestion tube and digested with 15 mL of concentrated HNO_3_ using a wet digestion method. After complete digestion, the solution was filtered and diluted to a defined volume with deionized water [43]. Arsenic concentrations were then measured using NexION 300X inductively coupled plasma mass spectrometry (ICP–MS; PerkinElmer, Waltham, MA, USA).

### 4.10. Statistical Analysis

All data in this study were statistically analyzed using Excel 2019 and IBM SPSS 19, and graphs were visualized using SigmaPlot 14.

## 5. Conclusions

In summary, the successful remediation of arsenite-contaminated soil using *B*. *contaminans* ZCC in combination with biochar is attributed to a multi-layered, complementary, synergistic mechanism. This comprehensive mechanism can be summarized by focusing on three aspects: (1) Physicochemical improvement: Biochar, as a soil amendment, directly increases soil pH and reduces the bioavailability of arsenic, providing a low-toxicity environment for plants and microorganisms. At the same time, its porous structure and nutrient release capacity optimize the physical properties and fertility of the soil. (2) Microbial growth promotion and colonization enhancement: Biochar provided a physical refuge and nutrient source for *B*. *contaminans* ZCC, extending its survival in the soil and enhancing its colonization efficiency and biological activity in the rhizosphere. (3) Direct plant growth promotion and indirect stress reduction: *B*. *contaminans* ZCC directly promotes the growth of *P. vittata* by secreting auxins and other substances; simultaneously, the synergistic effect of biochar and *B*. *contaminans* ZCC creates a low-stress growth environment for the plant by reducing arsenic toxicity and activating the plant’s antioxidant defense system.

Ultimately, this “soil improvement-microbial enhancement-plant promotion” triadic synergy results in the overall effect of the “ZCC + biochar + *P. vittata*” combined remediation system being far greater than the sum of the individual effects, providing an efficient, environmentally friendly, and widely applicable new strategy for the phytoremediation of arsenic-contaminated soils. Future research could further explore the specific signaling pathways of interaction between *B*. *contaminans* ZCC and the roots of *P. vittata* at the molecular level and conduct long-term field-scale experiments to verify the stability and durability of this technology in the remediation of actual contaminated sites.

## Figures and Tables

**Table 1:** Effects of biochar and *B*. *contaminans* ZCC co-application on the pH of soil polluted by arsenic.

Treatment	pH
CK	6.73 ± 0.23 c
ZCC	6.98 ± 0.20 b
1%BC	7.08 ± 0.16 b
1% BC + ZCC	7.02 ± 0.16 b
5% BC	7.56 ± 0.09 a
5% BC + ZCC	7.50 ± 0.15 a

Note: Values are mean ± standard deviation (*n* = 6). Different letters following the data in the same column indicate significant differences at the level of *p* < 0.05 (Duncan’s new multiple range test).

**Table 2:** Effects of combined application of different biochar ratios and *B. contaminans* ZCC on the growth of *P. vittata*.

Treatment	Total Plant Fresh Weight (g)	Total Plant Dry Weight (g)	Height (cm)
CK	12.94 ± 2.43 d	1.94 ± 0.36 d	19.25 ± 1.39 d
ZCC	17.66 ± 1.70 c	2.42 ± 0.51 c	22.24 ± 1.38 c
1% BC	16.97 ± 0.54 c	2.58 ± 0.38 c	19.68 ± 0.85 d
1% BC + ZCC	29.54 ± 2.64 b	4.32 ± 0.21 a	27.11 ± 1.74 a
5% BC	28.50 ± 1.48 b	3.79 ± 0.32 b	25.11 ± 0.57 b
5% BC + ZCC	33.14 ± 3.16 a	4.47 ± 0.38 a	28.47 ± 1.19 a

Note: Values are mean ± standard deviation (*n* = 6). Different letters following the data in the same column indicate significant differences at the level of *p <* 0.05 (Duncan’s new multiple range test).

**Table 3:** Effects of different biochar ratios combined with *B. contaminans* ZCC on arsenic content in *P. vittata* plant.

Treatment	Content (mg kg^−1^)
CK	15.83 ± 3.28 d
ZCC	22.42 ± 4.85 c
1% BC	26.86 ± 5.80 c
1% BC + ZCC	38.48 ± 3.25 b
5% BC	37.02 ± 2.60 b
5% BC + ZCC	44.42 ± 6.14 a

Note: Values are mean ± standard deviation (*n* = 6). Different letters following the data in the same column indicate significant differences at the level of *p <* 0.05 (Duncan’s new multiple range test).

**Table 4:** Basic physicochemical properties of the experimental soil.

Property	pH	OC (g kg^−1^)	TN (g kg^−1^)	TP (g kg^−1^)	TK (g kg^−1^)	AN (mg kg^−1^)	AP (mg kg^−1^)	AK (mg kg^−1^)	As (mg kg^−1^)
Value	6.90 ± 0.20	5.29 ± 0.22	0.49 ± 0.02	0.42 ± 0.05	27.50 ± 3.10	34.00 ± 3.85	29.60 ± 3.28	46.00 ± 5.46	24.16 ± 2.52

Note: OC—Organic carbon; TN—total nitrogen; TP—Total phosphorus; TK—Total potassium; AN—Alkaline nitrogen; AP—Available phosphorus; AK—Available potassium; As—arsenic. Values are mean ± standard deviation (*n* = 6).

## Data Availability

The data presented in this study are available on request from the corresponding author. The data generated in this study are primarily comprised of routine soil chemical properties and plant growth and physiological parameters. These data have been presented in the manuscript as means ± standard deviations in the figures and tables. As the study did not involve raw sequencing data, genomic information, or species identification data that require public archiving, the original datasets were not deposited in a public repository. However, the corresponding author will gladly provide the relevant data upon reasonable request for academic purposes.
